# Poisoning by Carbonic Acid

**Published:** 1847-05

**Authors:** 


### POISONING BY CARBONIC ACID.

A short time since, two negro men in Tennessee fell asleep upon the stack of an iron furnace filled with charcoal, which had been two or three days burning. In four or five hours they were found in a state of insensibility, but a trough of cold water being at hand, they were plunged into it, and, in the course of fifteen or twenty hours, restored to their senses, a little aqua ammonia having in the mean time been administered.

In the same State, a few years ago, a man went into his well in August, the water having given out. He fell down almost immediately, and his wife, having given the alarm, descended to his relief. A black man who arrived in a minute or two saw them both lying insensible at the bottom of the well, and running down was himself soon overpowered. The white man was taken out as quickly as possible, but never breathed afterwards. His wife, who was next removed, survived seven days, but ultimately died with typhoid symptoms. The negro was about fifteen minutes in the well; he recovered, and is still alive, but was hemiplegic for ten months, and even now has not the full use of his left side.

April 22d, 1847.

